# The Involvement of PPARs in the Peculiar Energetic Metabolism of Tumor Cells

**DOI:** 10.3390/ijms19071907

**Published:** 2018-06-29

**Authors:** Andrea Antonosante, Michele d’Angelo, Vanessa Castelli, Mariano Catanesi, Dalila Iannotta, Antonio Giordano, Rodolfo Ippoliti, Elisabetta Benedetti, Annamaria Cimini

**Affiliations:** 1 Department of Life, Health and Environmental Sciences, University of L’Aquila, 67100 L’Aquila, Italy; andrea.antonosante@gmail.com (A.A.); dangelo-michele@hotmail.com (M.d.); castelli.vane@gmail.com (V.C.); Mariano.catanesi86@gmail.com (M.C.); iannottadalila@gmail.com (D.I.); rodolfo.ippoliti@univaq.it (R.I.); elisabetta.benedetti@univaq.it (E.B.); 2 Sbarro Institute for Cancer Research and Molecular Medicine, Department of Biology, Temple University, Philadelphia, PA 19122, USA; giordano12@unisi.it; 3 Department of Medicine, Surgery and Neuroscience, University of Siena, 53100 Siena, Italy; 4 National Institute for Nuclear Physics (INFN), Gran Sasso National Laboratory (LNGS), 67100 Assergi, Italy

**Keywords:** nuclear receptors, energy metabolism, cancer metabolism

## Abstract

Energy homeostasis is crucial for cell fate, since all cellular activities are strongly dependent on the balance between catabolic and anabolic pathways. In particular, the modulation of metabolic and energetic pathways in cancer cells has been discussed in some reports, but subsequently has been neglected for a long time. Meanwhile, over the past 20 years, a recovery of the study regarding cancer metabolism has led to an increasing consideration of metabolic alterations in tumors. Cancer cells must adapt their metabolism to meet their energetic and biosynthetic demands, which are associated with the rapid growth of the primary tumor and colonization of distinct metastatic sites. Cancer cells are largely dependent on aerobic glycolysis for their energy production, but are also associated with increased fatty acid synthesis and increased rates of glutamine consumption. In fact, emerging evidence has shown that therapeutic resistance to cancer treatment may arise from the deregulation of glucose metabolism, fatty acid synthesis, and glutamine consumption. Cancer cells exhibit a series of metabolic alterations induced by mutations that lead to a gain-of-function of oncogenes, and a loss-of-function of tumor suppressor genes, including increased glucose consumption, reduced mitochondrial respiration, an increase of reactive oxygen species, and cell death resistance; all of these are responsible for cancer progression. Cholesterol metabolism is also altered in cancer cells and supports uncontrolled cell growth. In this context, we discuss the roles of peroxisome proliferator-activated receptors (PPARs), which are master regulators of cellular energetic metabolism in the deregulation of the energetic homeostasis, which is observed in cancer. We highlight the different roles of PPAR isotypes and the differential control of their transcription in various cancer cells.

## 1. Introduction

Mammalian cellular activities require a significant energy source, which is produced by specific mechanisms involved in the regulation of cellular energy homeostasis. The correct balance between catabolic and anabolic pathways strongly influence cellular fate, since they are involved in biochemical reactions that drive ATP (adenosine triphosphate) production/consumption. Oxidative glucose metabolism by OXPHOS (oxidative phosphorylation) produces up to 36 ATP per mole of glucose, whereas non-oxidative glucose metabolism by glycolysis results in two ATP per mole of glucose [1]. Hence, oxygen availability provides an optimal cellular condition to produce high levels of energy, while hypoxia determines a less efficient cellular condition in which the cells prefer to use glycolysis to produce energy. Another way to meet cellular energy demands is lipid metabolism by the peroxisomal and mitochondrial β-oxidation of fatty acids (FAs), which provides energy in the form of redox potential [2,3]. Regarding lipid metabolism, many cell types present cytosolic lipid deposits, also called lipid droplets (LDs). These are dynamic organelles that contain triacylglycerols (TAGs) and cholesteryl esters, and present several functions such as reducing lipotoxicity, lipid storage, and lipid metabolism, and they are directly involved in cellular physiology [4,5,6,7]. Unlike normal cells, cancer cells exhibit uncontrolled proliferation that needs energy metabolism adjustments in order to ensure their cell growth and division. The high proliferation rate in tumor cells leads to significant metabolic changes that are closely related to the environmental conditions and genetic/epigenetic characteristics of the tissue from which tumor arises. To safeguard their survival, cancer cells metabolically switch from less efficient energy pathways to higher performing energy pathways in order to cope with the considerable energy demands of tumor bulk. Meanwhile, neoplastic cells show altered glucose and lipid metabolism in association with unstable OXPHOS and glutamine metabolism; accordingly, PPARs play a key role in regulating these metabolic switch events. Therefore, our purpose in this review is to describe recent observations concerning the pivotal role of PPARs in promoting or preventing the characteristic metabolic switch that provides the energy for tumor survival. The main metabolic mechanisms adopted by tumor cells that are under the control of PPARs will be briefly described below.

### 1.1. Glucose Metabolism and OXPHOS in Cancer Cells

Although in normoxia, healthy cells use the degradation of glucose to pyruvate and later the TCA (tricarboxylic acid) cycle to produce ATP, neoplastic cells prefer to use glycolysis to produce energy rather than oxidative phosphorylation. The first observation of this phenomenon is about 88 years old, when Otto Warburg noticed that tumor cells switch toward a glycolytic metabolism with high lactate production, even in aerobic conditions, and mitochondrial metabolism suppression. This metabolic adaptation is called “aerobic glycolysis” or the “Warburg effect” [8]. Despite aerobic glycolysis not being influenced by oxygen levels, in hypoxic conditions, tumor cells present an overexpression of the genes involved in the glycolytic pathway. Usually, in solid tumors, near the core, there is a hypoxic area, and this hypoxic environment supports glycolytic metabolism and provides chemotherapy resistance as well as an optimal niche for the maintenance of CSCs (cancer stem cells) [9,10,11]. It was also observed that many types of cancer cells (glioma, hepatoma, and breast) are able to obtain ATP from OXPHOS, and they can pass from a fermentative to an oxidative metabolism and vice versa, and glucose is directly involved in this switch [12,13,14,15]. On the other hand, tumor cells can perform a glucose-dependent suppression of mitochondrial respiration, which is called the “Crabtree effect” [16]. This effect is reversible and collaborates with the “Warburg effect” to ensure cancer cell survival, independently from the presence of oxygen [17]. In a recent study, using a mathematical computational model, Epstein and collaborators [18] explored the coexistence between glycolytic and oxidative pathways in cancer cells, starting from the assumption that cancer cells quickly need ATP, but at the same time need to maintain baseline levels of ATP, mainly during moments of apparent standby. Consequently, in relation to fluctuating energy demands and assuming that tumor cells exist in a heterogeneous environment, they can use a glycolytic pathway to produce ATP quickly in short-term energy requests; conversely, baseline levels of energy are obtained through OXPHOS [18]. In addition, although lactate is a waste product of aerobic glycolysis, it is recycled by subpopulations of cancer cells and directed toward the TCA cycle [19]. These evidences lead to the belief that there is cooperation between different types of cells within the tumor, which could be a key mechanism for tumor progression. Several genes involved in the glycolytic pathway regulate the adjustment of cancer cells to the metabolic switch; some of them are oncogenes. Among them, PI3K/Akt signaling induces the expression of proteins related to glucose transport (GLUTs) in association with high hexokinase II (HKII) activity. HKII is able to bind to the voltage-dependent anion channel (VDAC) on the outer mitochondrial membrane to protect cells from apoptosis [20,21]. Moreover, altered c-Myc (cancer-myelocytomatosis) regulation affects the expression of the genes that are related to glutamine metabolism and aerobic glycolysis (HKII, lactate dehydrogenase (LDH), pyruvate kinase isoenzyme M2 (PKM2), phosphofructokinase 1 (PFK1), and GLUT1) [21,22], and PKM2 plays a central role in the shift of cellular metabolism to aerobic glycolysis in cancer cells. PKM2 is the specific isoform that is mainly expressed in tumor cells [23]. Whereas PKM1 is a constitutively active tetrameric enzyme, the 22 amino acid differences in PKM2 create a fructose 1,6-bisphosphate (FBP) binding pocket that renders it dependent on the allosteric binding of FBP for formation of an active tetramer. PKM2 activity is more flexible than PKM1 activity, which is why PKM2 is more suitable to guarantee the metabolic switch in cancer cells. In addition, PKM2 presents a low activity index, probably allowing the storage of glycolytic metabolites to ensure macromolecule biosynthesis [24]. In this context, the hypoxic environment provides an additional incentive to trigger the transcription of genes linked to the Warburg effect, and they are directly under the transcriptional control of hypoxia inducible factor-1α (HIF-1α) [25]. However, aerobic glycolysis is also essential for the macromolecule biosynthesis, in order to provide the structural components for cell proliferation. An increased flux of pyruvate provides the carbon source for the anabolic process, such as the de novo synthesis of nucleotides, lipids, and proteins. At the same time, the synthesis of macromolecules in cancer cells is necessary to produce reducing equivalents, such as NADH (nicotinamide adenine dinucleotide H) and NADPH (nicotinamide adenine dinucleotide phosphate H); in turn, they are essential for ensuring glucose metabolism, biosynthesis, and the degradation of macromolecules [26].

### 1.2. Lipid, Cholesterol, and Glutamine Metabolism in Cancer Cells

Fatty acids synthesis is typically reactivated in cancer cells by the upregulation of lipogenic enzymes to provide monomeric components for membrane building, lipid signaling, and post-translational protein modification [27]. Breast and prostate cancer show an increased expression of fatty acids synthase (FAS) and enzymes involved in the elongation of very long-chain fatty acids such as ELOVL1-7 (elongation of very long chain fatty acids protein 1-7) [28,29]. The stability and fluidity of cellular membranes are cholesterol-dependent, and lipid rafts (which are involved in the regulation of intracellular transduction signals) are mainly composed of cholesterol [30]. Furthermore, the mevalonate pathway (MVA), which is responsible for cholesterol synthesis, is linked to the production of intermediates that are crucial for post-translational modifications of Rho, Ras, and other small GTPase (isoprenylation, farnesylation, and genarylation) [31]. Interestingly, statins, which are drugs that are used to decrease plasma cholesterol levels in hypercholesterolemic conditions, inhibit HMG-CoA (3-hydroxy-3-methyl-glutaryl-coenzime A) reductase (HMGCR); this is the rate-limiting step of MVA. In support of the lipid importance in tumors, it was demonstrated that statins are able to decrease the proliferative index in breast cancer and acute myeloid leukemia cells, and make colorectal cancer cells more sensitive to chemotherapy [32,33,34]. Moreover, prostate cancer cells showed high levels of cholesterol [35]. The excess quantity of LDs in cancer cells are further evidence that FAs and cholesterol accumulate in many types of cancer. Label-free Raman spectroscopy imaging of high-grade prostate cancer and metastasis revealed the accumulation of abnormal LDs associated with PTEN (phosphatase and tensin homolog) loss and PI3K/Akt activation [36]; similar evidences were observed in the breast cancer cell line [37] and colon cancer stem cells [38]. Meanwhile, in gliomas, a higher amount of LDs was directly proportional to the degree of tumor aggressiveness [39]. As previously mentioned, FAs derived from free triacylglycerides or intracellular deposits can be metabolized to produce energy in the form of redox fuel. The early phases of this process occur in cytoplasm (triglyceride and monoacylglycerol lipases), and the late phases occur in mitochondria and are called fatty acid β-oxidation (FAO), but can occur also in the lumen of peroxisomes. The end products of lipid decomposition, such as NADH, FADH_2_, and acetyl-CoA, are directed toward the TCA cycle; this is the reason why some non-glycolytic cancers, such as prostate cancer and large B-cell lymphoma, need FAO to meet their energetic demands [40,41,42,43]. In spite of this, even some glycolytic tumors, under certain conditions require FAO to produce energy [43], while in glioblastoma, FAO contributes to protect the cells from oxidative stress by upregulation of detoxification enzymes, such as glutathione (GSH) [44]. Unlike aerobic glycolysis, where a hypoxic condition increases glucose utilization, lipid biosynthesis is not encouraged by oxygen lack, resulting in lipid accumulation into LDs [45,46]. In this scenario, the carbon source to synthesize lipid compounds is supplied by glutamine; isocitrate dehydrogenase-1 (IDH1) activity releases citrate in the cytosol after carboxylation of glutamine-derived α-ketoglutarate [47,48,49]. Moreover, Ras oncogene together with hypoxia induces the pyruvate dehydrogenase kinase 1 (PDK1), which in turn inhibits pyruvate dehydrogenase (PDH) and forces cells to implement glutamine-dependent anaplerotic behavior [45,48,50]. This phenomenon restores the TCA cycle under specific conditions and highlights the key role of glutamine metabolism in cancer cell growth. Beyond anaplerotic involvement, glutamine catabolism provides nitrogen to synthetize the nucleotide glutathione, resulting in the major energy source in some transformed cells [51]. Cancer cells rely on glutamine uptake to ensure a further pathway to support their accelerated metabolism. Gao et al. [22] showed that c-Myc stimulates glutaminase (GLS) expression through the suppression of miR-23a/b, while the inhibition of Rho-GTPase by a small compound determines the reduction of glutaminase activity, which is dependent of NFκB (nuclear factor kappa-light-chain-enhancer of activated B cells) in breast cancer and B lymphoma cells [52]. In addition, DeBerardinis and collaborators [53] observed that glioblastoma cells performed aerobic glycolysis associated with elevated glutamine catabolism to obtain redox energy and TCA cycle intermediates in order to support biosynthetic activity, mainly FAs. Interestingly, highly invasive ovarian cancer cells showed more remarkable glutamine dependence than low-invasive ovarian cancer cells; this feature is related to glutamine-mediated STAT3 (signal transducer and activator of transcription 3) modulation [54].

## 2. PPARs

Peroxisome proliferator-activated receptors (PPARs) are ligand-activated transcription factors belonging to the nuclear hormone receptor superfamily. PPARα (NR1C1) was the first described as the receptor mediating peroxisome proliferation in rodent hepatocytes in 1990; later, two related isotypes, PPARβ/δ (NR1C2) and PPARγ (NR1C3) were found and characterized. PPARα is mainly expressed in tissues presenting high fatty acid catabolism activity, such as the liver, the heart, the brown adipose tissue, the kidney, and the intestine; it is also involved in regulating lipoprotein synthesis. Regarding PPARγ, there are two isoforms: γ1 and γ2, which are obtained by alternative splicing. Both isoforms act in the white and brown adipose tissue to promote adipocyte differentiation and lipid storage, while only PPARγ1 is expressed in other tissues, such as the gut or immune cells. PPARγ transcriptional targets are also involved in regulating inflammatory processes, the cell cycle, and glucose metabolism by improving insulin sensitivity; in fact, it is a useful target for type 2 diabetes therapy. PPARβ/δ is ubiquitously expressed, and it has important functions in the skeletal muscle, adipose tissue, skin, gut, and the brain, including fatty acid oxidation regulation, keratinocyte differentiation, and wound healing [55,56,57,58].

Ordinarily, PPARs are active at transcriptional levels only in presence of their specific ligands, and each ligand is able to trigger a specific PPARs response; conversely, some findings demonstrated the basal activity of PPARs in the absence of ligands [59]. Unlike the steroid hormone receptors (nuclear receptors class 1) that function as homodimers, PPARs (nuclear receptors class 2) are active when they heterodimerize with retinoid x receptors (RXR); then, each monomer binds a specific DNA sequence, called PPREs (peroxisomes proliferator response elements). PPREs are direct repetitions that are located in the promoter region of the target gene as single or multiple copies [58,60,61]. As mentioned above, specific PPARs transcriptional activities are strictly related to lipid ligand type, and consequently, a wide range of natural or synthetic lipids can bind to the LBD (ligand-binding domain) of PPARs. These ligands can be obtained from diet or intracellular signaling pathways, among which FAs from prostaglandins and leukotrienes, as well as synthetic ligands, are described. Peculiar fatty acid-binding proteins (FABPs) allow the ligand delivery toward the nucleus, where PPARs reside [62]. Long-chain unsaturated FAs, eicosanoids, and hypolipidemic drugs (fibrates) can activate the PPARα, while thiazolidinediones (TZDs) are able to active PPARγ and increase insulin sensitivity. In this regard, PPARs are considered important therapeutic targets, mainly for metabolic diseases [63,64]. Given their role as master regulators of cellular energy pathways and considering the metabolic alterations in tumor cells, PPARs’ modulation can be involved in the specific metabolic plan undertaken by neoplastic cells. The central debate is whether the transcriptional activity of PPARs promotes or hinders tumorigenesis and tumor progression. To date, research activity has yielded conflicting evidence in this regard. There are several evidences about the tumor suppression role of PPARα and PPARγ [65,66,67,68,69,70,71], but there are also several evidences about their cancer promotion activity [72,73,74]; instead, regarding PPARβ/δ, the majority of study conducted shows its oncogenic role [75,76,77]. Although PPARs can have a dual role that is oncogenic as well as oncosuppressive, their behavior is severely influenced by the tissue type from which the tumor arises and by the tumor microenvironment.

### 2.1. PPARα and Cancer Metabolism

The process of tumorigenesis can be described by a series of molecular features, among which the alteration of cellular metabolism has recently emerged. This metabolic rewiring fulfills the energy and biosynthetic demands of fast proliferating cancer cells and amplifies their metabolic reserves to survive and proliferate in the poorly oxygenated and nutrient-deprived tumor microenvironment. In these harsh environmental conditions, the deregulation of glucose and glutamine metabolism, alterations of lipid synthesis and FAO, and a complex rewiring of mitochondrial and peroxisomal function are required. However, mitochondria and peroxisomes display close relationships; in fact, it was recently reported that in glioblastoma, an increase of peroxisomes leads to the increase of mitochondria [78].

PPARα mainly regulates the gene expression of specific proteins that are involved in mitochondrial and peroxisomal functions, such as fatty acids’ β-oxidation, glucose metabolism, and fatty acid transport [56,58]. The relationship between gene transcription regulated by PPARα and tumor metabolism can determine oncogenic or oncosuppressive effects. PPARα activation and tumor suppression was reported in melanoma [79] and glioblastoma [80]; on the other hand, PPARα activation demonstrated a positive role in stimulating the proliferation of breast and renal carcinoma cell lines [81,82], while *PPARα-*null mice were insensitive to hepatic carcinogenesis induced by PPARα agonist [83].

Several evidences support the paradigm that tumors originate from cancer stem cells and/or cancer stem progenitor cells, namely, tumor initiating cells or cancer stem-like cells (CSCs). CSCs represent a small population of cancer cells that exhibit self-renewal and differentiation features similar to normal stem cells, although they differ in the regulation of their self-renewal pathways. Based on the CSC presence, they are responsible for tumor formation, progression, metastasis, and relapse, as well as drug resistance. Even if it is generally known that tumor cells, particularly CSCs, show glucose and lipid metabolism alterations, the specific metabolic pathways and their regulation are still poorly understood [11]. Due to their crucial roles in energetic metabolism, the PPARs have been investigated by many authors regarding their involvement in tumorigenesis, showing an upregulation of the α isotype in several tumors and CSCs.

Recently, we demonstrated decreased tumor proliferation with an alteration of glucose and lipid tumor metabolism, antagonizing PPARα by synthetic ligand (GW6471) in glioblastoma stem cells (GSCs) [84]. GSCs are responsible for drug resistance and relapse; they reside in intratumoral perivascular and necrotic/hypoxic niches, which provide the GSCs with the optimal environment to keep their stemness features. Hypoxia is associated with glioblastoma progression and plays a crucial role in stem cells’ biology. HIF proteins regulate the cellular response to hypoxia or variable oxygen concentrations by upregulating the genes related to tumor progression, angiogenesis, drug resistance, and the phenotype maintenance of GSCs. Between HIF proteins, HIF-1α triggers the expression of genes related to tumor metabolic switch, which in turn induces glucose uptake, glycolytic enzyme activity, lactate production, and the anaerobic production of ATP. However, it is also able to control synthetic pathways (fatty acids and glycogen synthesis), stimulating the expression of anabolic enzymes, which are related to glucose–glycogen conversion [11,85,86]. In addition, we demonstrated that glioblastoma and GSCs in hypoxic condition show higher levels of PPARα compared with the normoxic condition [87], while PPARγ levels are downregulated under hypoxia [84]. In GSCs, glycogen storage appeared more abundant in hypoxia than in normoxia, since hypoxic cells need glucose to quickly produce ATP through glycolysis, and the glycogen storages are essential to maintain this fast energetic process. Moreover, HIF-1α stimulates the expression of genes involved in glycogen synthesis, as glycogen synthase kinase 3β (GSK3β). When GSK3β is phosphorylated at Ser 9, it is then inactive and unable to phosphorylate glycogen synthase, thus allowing the start of the anabolic process [88].

**Figure 1:**
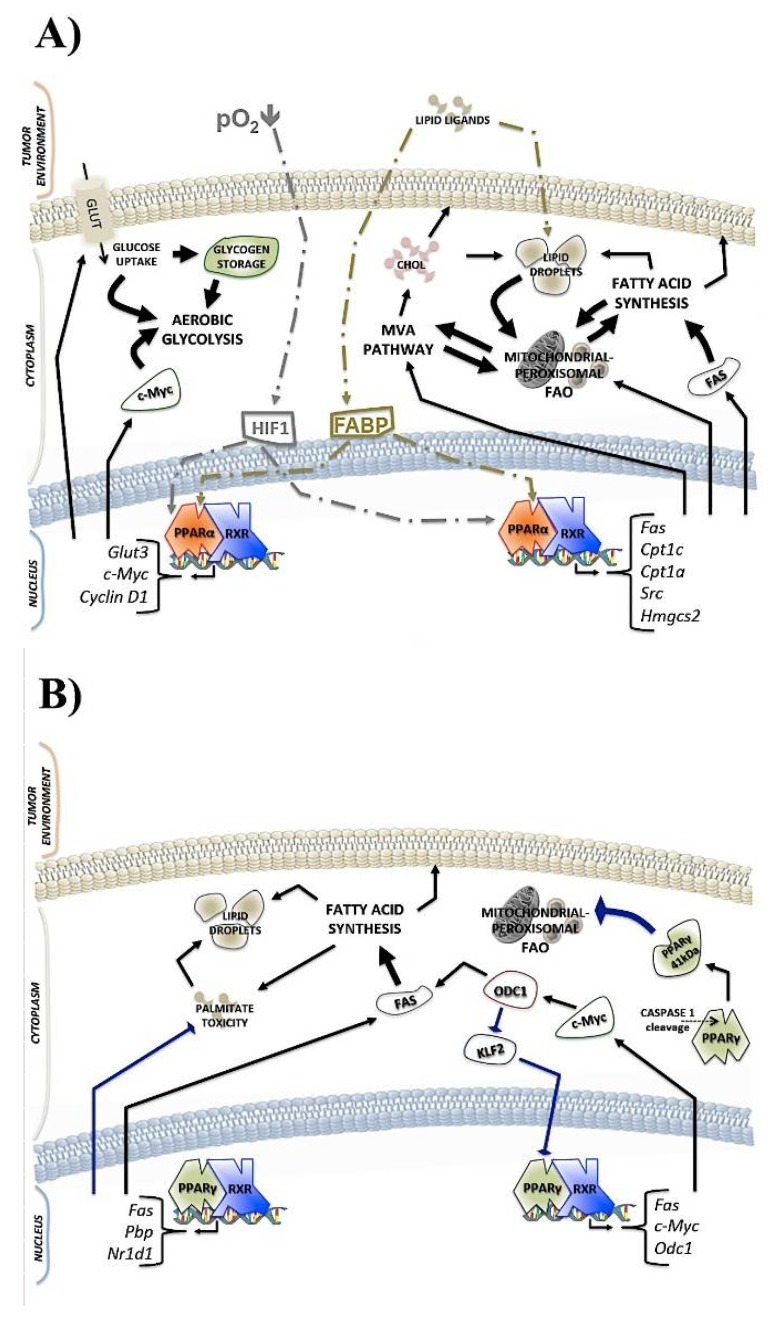

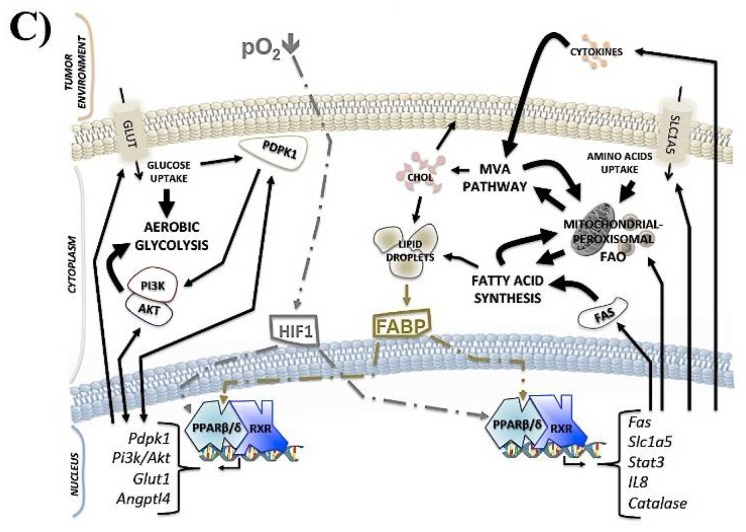
Schematic representation of PPARs-dependent oncogenic metabolic pathways highlighted in this review. The representation concerns the metabolic mechanisms that are activated/inhibited in tumor cells under the transcriptional control of PPARs. These hypotheses of molecular mechanisms are based on evidence obtained by different cancer types. For each PPAR isotype, the specific activated/inhibited metabolic pathways are reported together with some of the PPARs’ target genes. (**A**) Hypoxia-inducible factor-1 (HIF-1) can active PPARα, which in turn activates the transcription of specific genes resulting in high glycolysis, high glycogen storage, and high proliferation rate (glucose transporter 3 (GLUT3), c-Myc, and cyclin D1). However, PPARα activation is also related to the induction of fatty acid oxidation (FAO) by upregulation of carnitine palmitoyltransferase 1 (CPT1). In addition, PPARα induces fatty acid synthesis by upregulation of fatty acid synthase (FAS) enzymes. It is noteworthy that mitochondrial 3-hydroxy-3-methylglutaryl-CoA synthase (HMGCS2) is upregulated by PPARα; besides, HMGCS2 can form a heterodimeric complex with PPARα to induce Src expression. The phosphorylation of Src triggers the mevalonate (MVA) pathway, resulting in high levels of cholesterol (CHOL). Lipid components and cholesterol are useful for membrane synthesis, and their large amounts are confined in lipid droplets. Extracellular lipids and some intracellular lipids (from lipid droplets) can be PPARα ligands; they are delivered to the nucleus by fatty acid binding protein (FABP). (**B**) PPARγ transcriptional activity activates some proteins related to fatty acid synthesis, such as FAS, c-Myc, PBP (PPARγ-binding protein), NR1D1 (nuclear receptor subfamily 1, group D, number 1), and ODC1 (ornithine decarboxylase 1). ODC1 is able to inhibit krüppel-like factor 2 (KLF2), which in turn is unable to inhibit PPARγ. Other PPARγ-dependent mechanisms are able to reduce palmitate toxicity by confining it into lipid droplets. Moreover, PPARγ 41 kDa fragment, which is derived from caspase 1 cleavage, is able to inhibit FAO. (**C**) PPARβ/δ stimulates glycolysis by the overexpression of GLUT1, angiopoietin-like 4 (ANGPTL4), phosphoinositide-dependent protein kinase 1 (PDPK1), and PI3K/Akt; likewise, PDPK1 and PI3K/Akt can activate PPARβ/δ expression. Fatty acid synthesis and FAO are activated by PPARβ/δ transcriptional activity on FAS and SLC1A5 (solute carrier 1 A5) genes. SLC1A5 is linked to the uptake of amino acids; thus, anaplerosis is also positively affected by PPARβ/δ. Anaplerosis also supports FAO. Interesting, PPARβ/δ upregulates cytokines expression; for example, interleukin 8 (IL8) and cytokines in concert with PPARβ/δ induce STAT3 overexpression. The MVA pathway is a downstream process triggered by STAT3. The thin black continuous lines with arrows indicate upregulation events. The thick black continuous lines with arrows indicate a stimulation of the metabolic pathway. The thin blue continuous lines with bars indicate inhibition events. The thick blue continuous lines with bars indicate the inhibition of a metabolic pathway. The HIF-1-mediated upregulation of PPARs is represented by a grey dash dot and arrow at the end, while FABP-mediated ligand-dependent activation of PPARs is represented by a gold dash dot and arrow at the end.

GW6471 treatment decreased the viability of GSCs, the number and size of neurospheres, and induced apoptosis, which was associated with low glycogen supplies. Increasing glycogen degradation was due to the upregulation of glycogen phosphorylase (GPBB) and downregulation of phosphorylated GSK3β at Ser 9. Furthermore, a decreased amount of GLUT3 and glucose uptake in hypoxic-treated GSCs have been reported. Moreover, it was also demonstrated higher amounts of LDs in cancer cells, mainly in the hypoxic environment, which is in line with previous evidence [21,39,87,89]. FABP7 (fatty acid binding protein 7) transports the fatty acids toward the nucleus; in the same way, it supplies the LDs to promote tumor growth [90,91], and it appears increased by hypoxia. GW6471 treatment induced the loss of LDs amounts, cholesterol supply, and the transcriptional activity of genes encoding for mevalonate pathway enzymes. However, FABP7 levels appeared decreased only in antagonist-treated hypoxic GSCs, since the inhibition of PPARα transcriptional activity in hypoxic GSCs adversely affects fatty acid and cholesterol amounts. The MVA pathway plays a central role in glioblastoma survival; besides, its inhibition by PPARα antagonist is linked to cell death and tumor suppression. This effect is similar to the downregulation of the MVA pathway together with the upregulation of PPARγ induced by statins. These results seem to emphasize the key role of PPARα in the metabolic switch that occurs in cancer hypoxic cells, such as GSGs. In harsh environmental conditions PPARα was upregulated and could result in metabolic directives to ensure energy for tumor cells. In this regard, the antagonist GW6471 was able to reduce the synthetic processes, such as glycogen synthesis and LDs biogenesis, which normally ensure fast-acting energy for cancer cells (as summarized in Figure 1A).

In another study, Abu Aboud et al. [82] used the same PPARα antagonist (GW6471) to treat two cell lines (Caki-1 and 786-O cell line) of renal cell carcinoma (RCC). They observed that PPARα levels were higher in high-grade RCC tissue compared with low-grade tissue, linking PPARα protein levels to RCC aggressiveness. High-grade RCC presents more energy demands than low-grade RCC, and therefore requires active fatty acid oxidation (FAO), which is regulated upstream by PPARα [92]. Both the antagonist and siRNA directed against the PPARα showed the capability of reducing c-Myc protein levels, which is likely by PPARα-mediated alteration of oncoprotein stabilization. This event was associated with the downregulation of cyclin D1/CDK4 and the G1/S transition block with cell cycle arrest in G0/G1 phase [93,94]. The authors hypothesized that the transcriptional activity of PPARα was inhibited, which was the reason why the renal carcinoma cells were unable to use FAO by converging on glycolysis to obtain energy. In fact, GW6471 effects were more pronounced in media with low glucose concentrations than media with normal glucose concentrations. Furthermore, 2-Deoxy-d-glucose (2-DG), which is an inhibitor of glycolysis, acted in synergy with GW6471 to induce tumor death. Regarding that, by blocking PPARα in the RCC cell line, the researchers demonstrated the reduction of cell viability with a marked reduction of c-Myc, cyclin D1, and CDK4 protein levels in synergy with glycolysis inhibition (as shown in Figure 1A).

It has been ascertained that the most of oncogenes are involved in the metabolic reprogramming of tumor cells [95,96]. Among them, cyclin D1, contrary to what has just been mentioned, was demonstrated to inactivate the PPARα-mediated gene expression of enzymes related to FAO in hepatocytes as well as hepatocellular and breast cancer-derived cell lines [97]. Previous evidences have demonstrated the role of cyclin D1 in the regulation of androgen receptors, estrogen receptors, thyroid hormone receptor, and PPARγ [98,99] in different cell types. Several pieces of evidence about the cyclin D1 regulation of cell metabolism, via the inhibition of PPARα transcription factor has been provided, while the overexpression of cyclin D1 induced the low expression of genes related to FAO. On the other hand, knockdown of cyclin D1 promoted FAO enzymes expression, but PPARα gene silencing weakened this effect. These results highlight the role of cyclin D1 in affecting FAO in a PPARα-dependent manner; for instance, a mitogen-stimulated cancer cell line showed low PPARα and FAO activity, indicating that the transition from a quiescent state to a proliferation state requires less energy from fatty acid [99]. Data reported in the paper of Kamarajugadda et al. [97] suggest that cyclin D1 blocks the binding of PPARα on the PPRE of specific FAO enzymes in a not clear way. At the same time, cyclin D1 could disturb the association of specific co-activators with PPARα, and then determine some changes in chromatin conformation; besides, cyclin D1 controls the expression of CBP/p300 [100,101].

Fatty acid synthase (FAS) is upregulated in a tumor of the urinary tract, such as RCC, and the downstream intermediates of fatty acid synthesis are endogenous ligands of PPARα, while the inhibition of FAS in the liver of mice provides rodents with PPARα dysfunction [102,103,104]. As mentioned above [82], a histological grade of RCC is directly linked to PPARα levels, and its inhibition leads to cell cytotoxicity, cell cycle arrest with glycolysis, and FAO deregulation. Recently, in RCC cell lines (Caki-1 and 786-O) and normal human kidney cells (NHK), it was reported that the inhibition of glycolysis triggered FAO and OXPHOS, even though PPARα inactivation reversed this metabolic pattern. Moreover, in normal cells, PPARα antagonist did not inhibit the glycolysis; conversely, in RCC, cell line glycolysis was attenuated, which was likely due to a difference of c-Myc protein levels between cancer cells and normal cells [105]. FAO can be considered an alternative metabolic pathway to produce energy when the glycolysis is obstructed. In fact, the RCC cell line showed increased levels of palmitate 24 h following 2-DG administration. Instead, co-administrations with GW6471 involve the decay of palmitate levels. Usually, fatty acid β-oxidation provided the acetyl-CoA groups to supply TCA cycle and OXPHOS, which in turn are more active with glycolysis inhibition. When RCC cell lines were treated with a combination of 2-DG and PPARα antagonist (GW6471), the OXPHOS activity levels showed no significant differences compared to the control cells, while GW6471 alone was able to impair oxidative phosphorylation, but not FAO. Therefore, PPARα antagonist adversely affected the levels of oncogene c-Myc in the RCC cell line, which is involved in the overactivation of protein related to glucose uptake and glycolysis. Most likely, PPARα controls glycolysis via c-Myc at least in RCC cell lines, and the simultaneous administration of 2-DG also induces FAO inhibition. This double effect is detrimental to the main metabolic pathways that are normally used by the RCC cell line [82,105].

Human hepatocellular carcinoma (HCC) tissue showed increased mRNA levels of the gene involved in FAO and glucose metabolism, among which PPARα, carnitine palmitoyl transferase 1A (CPT1A is the rate-limiting enzyme of FAO), glyceraldehyde 3-phosphate dehydrogenase (G3PDH), and the upregulation of cyclin D1 mRNA. Although increased levels of PPARα were associated with the deregulation of metabolic pathways that trigger carcinogenesis, there has not been evidence of HCC incidence in human patients who were exposed to peroxisome proliferators [106].

Regarding carnitine palmitoyl transferase enzyme, the possible regulatory role of PPARα-CPT1C axis in tumor proliferation and senescence was recently demonstrated [107]. As mentioned above, as CPT1A, CPT1C is also a rate-limiting enzyme in FAO, and the enzymatic reaction allows the acylation of a long fatty acid chain with subsequent entry into the mitochondria. In cancer cells, the CPT1 enzyme’s family is upregulated [108,109,110]. Moreover, it was identified as PPRE in the first exon of the CPT1B gene [111,112]. In order to investigate the possible relationships between CPT1 genes and PPARα, were performed some analyses on two different cancer cell lines, MDA-MB-231 (breast cancer cell line) and PANC-1 (pancreas cancer cell line) with knockdown or overexpression of the PPARα gene. Dual-luciferase reporter gene assays showed *CPT1C* active transcription by PPARα in association with cell proliferation and senescence interruption. The effects were completely different when the PPARα gene was depleted; an increase in senescence with low proliferation rate was observed, indicating that the CPT1C gene is regulated by PPARα. This is further evidence of the ability of PPARα to modulate cancer cell metabolism (see also Figure 1A) [107].

During carbohydrate deprivation, the cells can adopt ketogenesis to ensure lipid-derived energy; this process is essential for tumor initiation and metastasis [113]. Mitochondrial 3-hydroxy-3-methylglutaryl-CoA synthase (HMGCS2) belongs to the HMG-CoA family, and catalyzes the first enzymatic reaction in ketogenesis. Several proteins related to the ketogenesis pathway were overexpressed in prostate cancer cells [114], among which HMGCS2 was included; on this basis, some researchers demonstrated the direct interaction between PPARα and HMGCS2 [115], resulting in Src activation and the promotion of malignancy and invasion. This study demonstrated the correlation between the increased mRNA levels of HMGCS2 and poor clinical outcomes as well as grade malignancy in colorectal cancer (CRC) and oral squamous cell carcinoma (OSCC) tumor biopsy from affected patients. The demonstration of a direct interaction at the nuclear level between HMGCS2 and PPARα is interesting; besides, other analyses confirmed that the heterodimeric complex binds the *Src* promoter region and induced genes linked to tumor invasion (Figure 1A) [115].

Chronic lymphocytic leukemia (CLL) patients present poor clinical outcomes, and the most effective therapy is based on high dose of glucocorticoids (GCs) with or without monoclonal antibodies. Nevertheless, this therapeutic protocol is not curative, and is characterized by progressive tumor resistance to GCs [116]. Glucocorticoids have immunosuppressive effects, inhibiting glucose metabolism and increasing FAO in tissue under starvation condition. Tung et al. [117] found in CLL that primary culture from patient’s blood increased PPARα expression mediated by GCs with pronounced tumor dependence on FAO. Lipid oxidation ensures tumor survival, providing an alternative mechanism to the metabolic limitations dictated by GCs. PPARα antagonist impaired the tumor chemoresistance mechanism of GCs. Pyruvate kinase M2 (PKM2) activity was downregulated at the transcriptional and protein level by dexamethasone (DEX); despite this, acetate levels were kept constant, suggesting an increase in FAO activity linked to DEX. PPARα and PPARβ/δ mRNA levels were increased after DEX administration, while the downregulation of PKM2 occurred before the PPARα upregulation; it is likely that the nuclear receptor did not affect pyruvate kinase gene transcription. Nevertheless, the pyruvate dehydrogenase kinase 4 (PDK4) gene is under the transcriptional control of PPARα and PPARβ/δ; then, PDK4 phosphorylates and inhibits pyruvate dehydrogenase. Thus, pyruvate is useful for FAO rather than for OXPHOS [118]. Moreover, in order to understand the role of DEX in FAO and related chemoresistance triggering, the effects of DEX administration in association with FAO substrates were investigated. About that, CLL cells were co-cultured with OP-9-derived adipocytes in order to obtain an in vitro model in which lipids were derived from cells with an adipocyte phenotype. This model was used to mimic an in vivo tumor environment, where CLL cells are close to the adipocyte, and the high amount of lipids in the surrounding environment could improve tumor resistance to drugs by feeding FAO [119,120]. CLL showed greater resistance to DEX when cultured with adipocytes compared with CLL cells in serum-free media, and the effects were the same with conditioned media from an OP-9-derived adipocyte. These results highlight that lipids secreted from OP-9-derived adipocytes conferred chemoresistance. This experimental evidence demonstrated the direct involvement of PPARα in GCs tumor resistance, since it is upregulated by DEX and is a well-known FAO regulator; in addition, PPARα antagonists revoked these effects and sensitized CLL cells to DEX [117].

**Figure 2:**
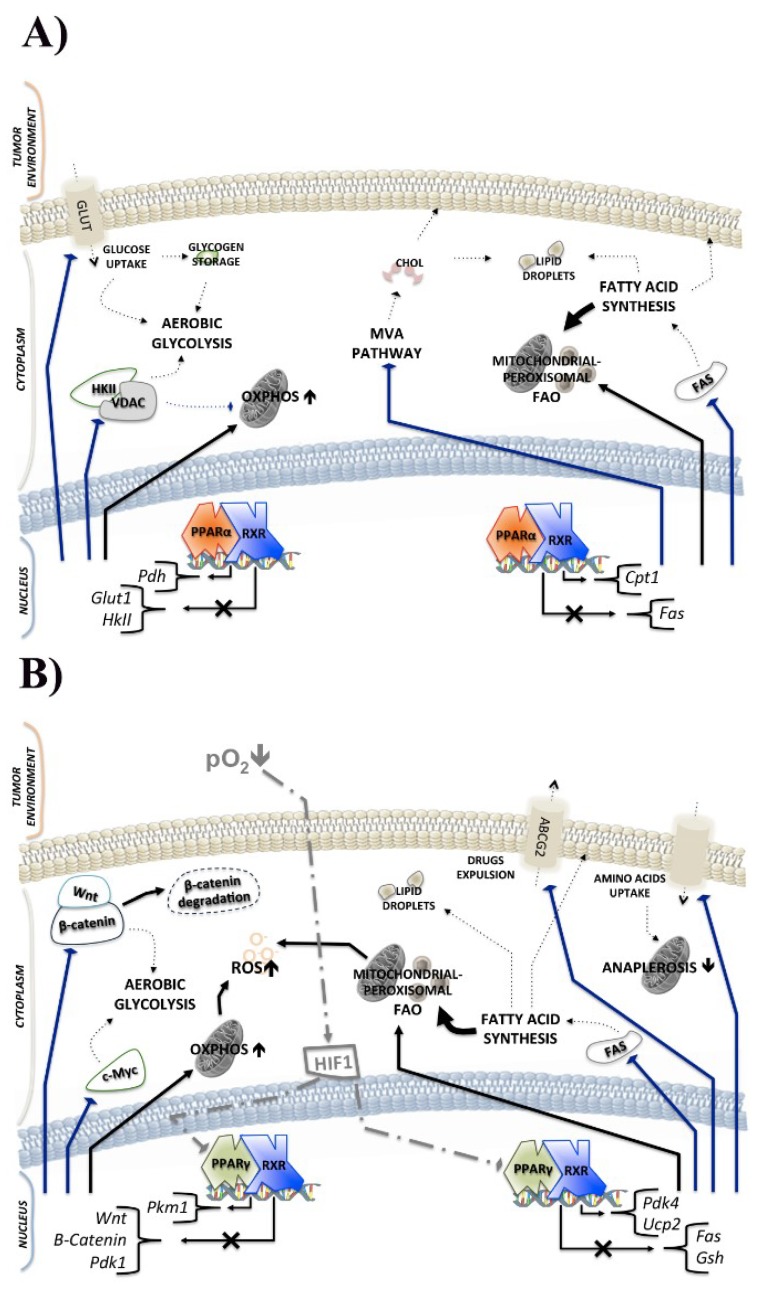

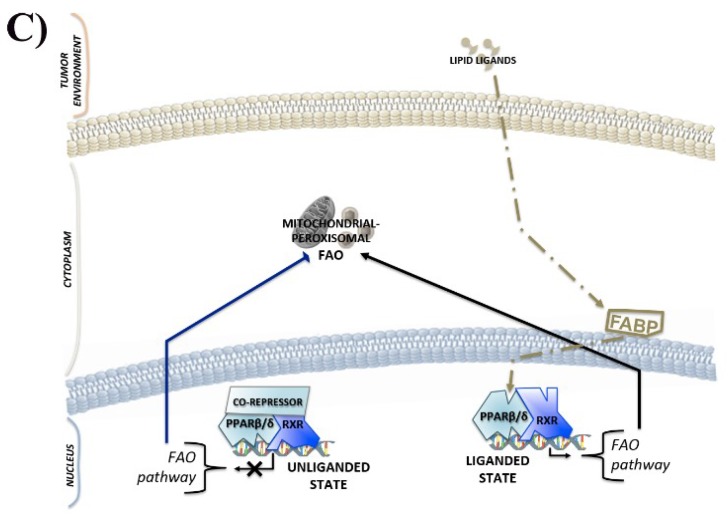
Schematic representation of PPARs-dependent oncosuppressive metabolic pathways highlighted in this review. The representation concerns the metabolic mechanisms that are activated/inhibited in tumor cells under the transcriptional control of PPARs. These hypotheses of molecular mechanisms are based on evidences obtained by different cancer types. For each PPAR isotype, the specific activated/inhibited metabolic pathways are reported together with some PPAR target genes. (**A**) Aerobic glycolysis is inhibited by the PPARα’s transcriptional repression of glucose transporter 1 (GLUT1) and hexokinase II (HKII) genes. Meanwhile, the complex between the voltage-dependent anion channel (VDAC) complex and HKII is destroyed by PPARα activity, thus adversely affecting glycolysis and increasing oxidative phosphorylation (OXPHOS). In addition, pyruvate dehydrogenase (PDH) is upregulated by PPARα to promote OXPHOS. Impairment in fatty acid synthesis by the downregulation of fatty acid synthase (FAS) and impairment of the mevalonate (MVA) pathway are due to effects adversely exerted by PPARα on specific target genes. Conversely, carnitine palmitoyl transferase 1 (CPT1) is upregulated by PPARα; this condition promotes fatty acid oxidation (FAO). Despite the reduced activity of fatty acid synthesis, FAO depletes insufficient lipid reserves and impairs cancer cells for life. (**B**) PPARγ downregulates the c-Myc/Wnt/β-catenin axis and stimulates β-catenin proteasome degradation. Further downregulation of pyruvate dehydrogenase kinase 1 (PDK1) and upregulation of pyruvate kinase isoenzyme M1 by PPARγ promotes OXPHOS and impairs aerobic glycolysis. Fatty acid synthesis, amino acid uptake, and anaplerosis are adversely affected by PPARγ activity in concert with increased levels of FAO. High FAO levels are related to the upregulation of PDK4 and mitochondrial uncoupling protein 2 (UCP2). Moreover, PPARγ activity negatively affects ATP binding cassette G2 (ABCG2) and prevents chemoresistance; this is associated with the high sensitivity of tumor cells to ROS, whose levels are increased through FAO and OXPHOS metabolic pathways. In addition, there is glutathione (GSH) downregulation, while hypoxia inducible factor-1 (HIF-1) is able to inhibit PPARγ activity. (**C**) In the absence of ligands, PPARβ/δ acts as a repressor, which is probably due to the strong interaction between PPARβ/δ/RXR heterodimer and a co-repressor. However, the repressor complex is able to downregulate the genes involved in FAO, this condition is abolished in the presence of exogenous or endogenous PPARβ/δ ligands. The thin black continuous lines with arrows indicate upregulation events. The thick black continuous lines with arrows indicate the stimulation of a metabolic pathway. The thin black dashed lines with arrows indicate a reduction activity of metabolic pathways. The thin blue continuous lines with bars indicate inhibition events. The thin blue dashed lines with bars indicate a reduction of the inhibition of the metabolic pathway. The HIF-1-mediated downregulation of PPARs is represented by a grey dash dotted line with a bar at the end, while the FABP-mediated ligand-dependent activation of PPARs is represented by a gold dash dotted line with an arrow at the end.

Contrary to what is stated, PPARα activity could be useful to counteract tumor progression in some tissue, as evidenced in melanoma [79]. In addition, PPARα is able to decrease the transcription of fatty acid synthesis genes and positively affect the transcription of FAO enzymes. In this regard, Chandran et al. reported the protective roles of the clofibrate, which is a PPARα agonist, in counteracting breast cancer inflammation and invasion [121]. The researchers used two triple negative breast cancer cell lines, SUM149PT and SUM1315MO2; the first from an invasive ductal carcinoma of a patient with inflammatory breast cancer, and the second from a highly invasive breast cancer specimen of a patient with skin metastasis. These two cell lines showed an increased expression of PPARα with respect to primary human mammary epithelial cells (HMEC). Clofibrate was able to reduce inflammation by decreasing the levels of COX-2 (cyclooxygenase-2) and 5LO (5-lipoxygenase) in association with the inhibition of growth tumor. Early events of cancer development require the upregulation of fatty acid synthesis, which is dramatically exacerbated during the late events of tumor progression [122]. FAS activity was attenuated by clofibrate, which in turn downregulated the expression of HMG-CoA synthase 2, acyl-CoA oxidase, and the sterol regulatory element binding protein 1c (SREBP-1c) gene. HMG-CoA synthase 2 and acyl-CoA oxidase are involved in the mevalonate pathway, while SREBP-1c is a transcription factor acting on sterol regulatory element DNA sequences. SREBP-1c (sterol regulatory element binding protein 1c) plays a key role in regulating de novo fatty acid synthesis, while its cognate SREBP-2 regulates the genes of the cholesterol metabolism [123]; SREBP’s pathway has a significant role in the de novo fatty acid synthesis of prostate cancer cells [124]. As reported by Chandran et al. [121], the activation of PPARα by clofibrate was able to impair the gene expression of SREBPs and reduce the NFκB and Erk1/2 (extracellular signal-regulated kinase 1/2) protein levels in breast cancer cells derived from high metastatic inflammatory tumor specimens. Conversely, in the same cancer cells, clofibrate was linked to the CPT-1a (first enzyme in FAO) upregulation (as reviewed in Figure 2A) [121].

Some evidence has indicated PPARα activation as a possible trigger of ineffective tumor metabolism. It was reported that the administration of fenofibrate (a PPARα agonist), on cell lines and a mouse model of oral cancer, supported hexokinase II and VDAC (voltage-dependent anion channel) dissociation. This event destabilizes the Warburg effect and provides a metabolic switch to OXPHOS. Furthermore, in these in vivo and in vitro oral cancer models, the activity of fenofibrate affected hexokinase II, PDH, and VDAC protein levels, as indicated in Figure 2A [125,126,127]. Recently, Huang and Chang [128] studied, through proteomic analysis, the differences between normal and cancer oral tissue from mice, relating it to enzymes involved in the Warburg effect. At the same time, they investigated the role of PPARα in the fibrate-dependent metabolic changes of the oral cancer cell line. Proteomic analyses were performed in a basic isoelectric point (pI) range, because the enzymes of glycolysis, the TCA cycle, and OXPHOS show mainly alkaline pI [128,129]. Seven proteins showed decreased levels in tumor tissue compared with normal tissue; they were triosephosphate isomerase and pyruvate dehydrogenase E1 component subunit beta for glycolysis, IDH3 and aconitate hydratase for the TCA cycle, NADH dehydrogenase [ubiquinone] 1 alpha subcomplex subunit 10 and cytochrome c1 for the respiratory chain. Considering oral cancer cells’ dependence on the Warburg effect, the researchers evaluated the effect induced by fibrate. PPARα activation induced the reduction of hexokinase II protein levels, ATP levels, and enhanced PDH activity, alongside reducing cell viability. Interestingly, they observed a significant increase in TCA cycle metabolites after fenofibrate administrations in primary cell culture from mouse tongue tumor tissue. Probably, PPARα agonist increased PDH activity; accordingly, pyruvate was decarboxylated to acetyl-CoA, and TCA cycle was encouraged. Otherwise, fenofibrate could increase FAO, resulting in high acyl group levels that are useful for TCA cycle reactions (Figure 2A) [127,128].

Regarding the Warburg effect and related aerobic glycolysis, the repression activity of PPARα on the GLUT1 gene with reduced glucose uptake was reported; these evidences were obtained in different cancer cell lines (HCT-116, SW480, MCF-7, and HeLa) (as indicated in Figure 2A) [71].

### 2.2. PPARγ and Cancer Metabolism

Several cell types express PPARγ, which is involved in different mechanisms that are essential to sustain normal cell life. Adipose tissue, liver tissue, muscle, brain, and immune cells (mainly macrophages) require PPARγ activation to meet energy demands and regulate glucose and lipid metabolism, insulin sensitivity, and cell fate. PPARγ plays a key role in adipocytes and the differentiation of macrophages [130,131,132]. As previously mentioned for PPARα, as well as for PPARγ, there have been several demonstrations about its role in tumorigenesis, some of them related to the antiproliferative effects of PPARγ activation, such as in breast [133], hepatic [134], lung [135], and colorectal cancer [136]. Moreover, PPARγ activation negatively affects the epithelial mesenchymal transition (EMT) [137]. However, there is other proof of the tumorigenic potential of PPARγ activation, such as in colorectal cancer [138,139,140], breast cancer [141,142], and urological cancer [143]. Both roles of PPARγ are strictly tumor tissue-dependent and tumor microenvironment-dependent.

Several types of epithelial cancers show a common feature: deregulation of the Wnt/β-catenin pathway, resulting in the upregulation of enzymes related to aerobic glycolysis. The availability of Wnt ligands triggers the nuclear translocation of the β-catenin, where it is able to bind specific target genes, including pyruvate dehydrogenase kinase (PDK), monocarboxylate lactate transporter-1 (MCT-1), c-Myc, cyclin D1, and COX-2. Without Wnt ligands, β-catenin is phosphorylated and then demolished by proteasome. In this view, PPARγ downregulation is associated with Wnt/β-catenin upregulation; on the other hand the inhibition of Wnt/β-catenin is mediated by PPARγ activation (see also Figure 2B). Accordingly, it is not inconceivable to think about a mechanism of interconnection between Wnt/β-catenin and PPARγ, in which each one is able to prevent the pathway of the other, as already demonstrated [144]. PDK1 acts as a phosphorylating pyruvate dehydrogenase, and then pyruvate is transformed in lactate by the activation of lactate dehydrogenase. Meanwhile, MCT-1 is involved in lactate secretion outside the cytoplasm. These two events enable improving the angiogenesis and biosynthesis of macromolecules, thus providing a unique and favorable tumor microenvironment [21]. In this context, PPARγ suppresses *PDK1* gene transcription, resulting in an ineffective Wnt/β-catenin pathway (Figure 2B) [145].

Studies conducted on PPARγ agonist or with PPARγ overexpressing cells, support the idea that PPARγ activation is useful to counteract tumor progression; in fact, thiazolidinediones (TZDs) show the ability to contain tumor growth in vitro and in vivo models of lung cancer. In addition, it was reported that the overexpression of PPARγ in a group of non-small lung cancer cells and its activation affect some genetic pattern underlying the tumor metabolic demands [146]. Srivastava and collaborators [135] reported in two lung adenocarcinoma cell lines (NCI-H2347 and NCI-H1993) that PPARγ activation compromised glucose, fatty acid, and glutamine metabolism, which are associated with increased ROS (reactive oxygen species) and hypophosphorylated RB (retinoblastoma protein). Dephosphorylated RB is opposed to the cell cycle progression. Unlike what was previously mentioned, in this work, the researchers found an upregulation of PDK4 expression by pioglitazone, and the central role of PDK4 in inducing the metabolic switch from glucose oxidation to fatty acid oxidation was suggested. PDK4 knockdown abolished the effect induced by pioglitazone related to RB hypophosphorylation and ROS levels; simultaneously, the same results were achieved in cell lines and xenograft mice models by inhibiting FAO with chemical compounds. These results suggested that PDK4 upregulation, by pioglitazone, compromised glucose utilization and triggered FAO with a subsequent increase of ROS levels, which in turn induced RB hypophosphorylation. Moreover, the researchers reported alterations in glutamine metabolism, an impairment of glutaminolysis, and downregulation of reduced glutathione (GSH) levels; therefore, tumor cells were unable to carry out ROS detoxification processes (as reported in Figure 2B) [135].

One common feature of several tumors such as non-small cell lung cancer (NSCLC) is resistance to radiation and chemotherapy, but the specific mechanisms are not entirely understood. However, it is well-known that hypoxia supports the malignancy and expression of ATP-binding cassette (ABC) transporters, which drive chemotherapeutic agents outside the cells [147,148]. The hypoxic condition is also combined with the downregulation of mitochondrial uncoupling protein 2 (UCP2) in NSCLC cells, as highlighted in a recent work [149]. UCP2 is a mitochondrial protein that is involved in the detoxification process by reducing ROS levels, because the cells are more sensible to superoxide anion released after proton force development by the electron transport chain. Moreover, a double role was suggested for UCP2: the reduction of ROS levels, and the metabolic regulation of glycolysis, fatty acid, and glutamine oxidation [150]. Downregulation of UCP2 by hypoxia was associated with PPARγ repression, the upregulation of the ABC transporter and ATP binding cassette G2 (ABCG2), and an increase of aerobic glycolysis and chemoresistance. HIF-1 was directly involved in PPARγ and FAO downregulation; this condition negatively affected the *UCP2* transcription. Conversely, glucose consumption was stimulated and established a progressive increase of ROS in concert with ABCG2 upregulation, as indicated in Figure 2B [149,151]. 

Several studies show the ability of ATRA (all-trans retinoic acid) to induce the differentiation of some myelocytic cell lines (HL-60, U937, and NB4) into mature phagocytic cells. ATRA administration is useful for the therapy of acute promyelocytic leukemia (APL), but the permanent administration of ATRA causes high resistance at differentiation, because there is overexpression of cytosolic retinoic acid binding proteins [152,153]. In this regard, the association between ATRA and PPARγ ligands was demonstrated to be synergistic in the differentiation effect on myelocytic leukemia cell lines [130]. The synergistic effect also concerned the enhancement of lipogenesis, as evidenced in the NB4 cell line by an accumulation of lipid droplets. Therefore, an induction of differentiation by ATRA and pioglitazone results in a high activity of triacylglycerol synthesis in human myelocytic leukemia cell lines [154].

An induction of PPARγ activity and concomitant autophagic cell death in human chronic myeloid leukemia (CML) cell lines (K562 and KCL-22) was reported by Shinohara et al. [155]. By docking analysis, they observed that anti-cancer fatty-acid derivative, called AIC-47, was able to bind PPARγ, making it transcriptionally active, and indirectly reducing c-Myc protein levels, since PPARγ activation is related to the proteasome degradation of β-catenin, as already mentioned [144]. Other interesting results also demonstrated the involvement of AIC-47/PPARγ in the deregulation of the glycolytic pathway. In fact, the upregulation of c-Myc is a cause and a consequence of aerobic glycolysis in tumor cells. As previously demonstrated, c-Myc can induce the overexpression of three heterogeneous nuclear ribonucleoproteins (hnRNPs), and, in turn, they can suppress the alternative splicing of pyruvate kinase isoenzyme M1 (PKM1), which is the less present isoform in cancer cells. Unlike other isoforms of PK that need allosteric regulation to be active, PKM1 is a tetrameric stable and active enzyme; for this reason, cancer cells prefer to use PKM2 for their metabolic purposes. PKM2 shows slow activity in cancer cells, because it also allows the biosynthetic pathways; consequently, in cancer cells, the PKM1/PKM2 ratio is low and c-Myc-dependent [23,24,156]. PPARγ activation AIC-47-dependent induced c-Myc downregulation, resulting in β-catenin inactivation with an increase of the PKM1/PKM2 ratio and the metabolic switch from glycolysis to the TCA cycle; simultaneously, ROS levels increase, which results in autophagy induction (Figure 2B) [155].

Survival in hepatocellular carcinoma (HCC) patients is related to the expression patterns of some genes, including ODC1 (ornithine decarboxylase 1). Its overexpression is associated with reduced patients survival [157]. The OCD1 enzyme catalyzes the first reaction in the biosynthesis pathway of polyamine; its mRNA and protein levels are increased together with c-Myc activity in HCC tissue compared with normal tissue [158]. An impairment of OCD1 expression by gene silencing was related to cell cycle interruption and apoptotic cell death; besides, phenotypic alterations occurred through a characteristic deregulation of 119 genes. Among them, it was interesting that the downregulation of PPARγ gene and lipogenesis were both linked to the upregulation of KLF2 (krüppel-like factor 2) oncogene. It was reported that the siRNA of ODC1 gene induced the upregulation of the KLF2 gene, which, in turn, negatively affected PPARγ expression, thus causing a downregulation of lipogenic enzymes, such as FAS and ACC2 (acetyl-CoA carboxylase 2), as already highlighted; see also Figure 1B [159,160].

Regarding de novo fatty acid synthesis, in ERBB2 (erythroblastic oncogene B)-positive breast cancer cells, a remarkable amount of lipid droplets was observed. ERBB2 cells assumed this metabolic behavior under the transcriptional control of PPARγ, and the inhibition of PPARγ decreased tumor cell viability. By RNA interference screening, some genes that are required for fatty acid metabolism and tumor cell survival were identified [161]. Within this group of genes, two were associated with PPARγ activity: PBP (PPARγ-binding protein) and NR1D1 (nuclear receptor subfamily 1, group D, number 1). Both were identified as activators of PPARγ expression; it is likely that PBP was a co-activator, and NR1D1 was the target gene [162]. The gene sequence of *PBP* and *NR1D1* are located in the ERBB2 amplicon, and in breast cancer, mutations in this gene locus are linked to high lipid synthesis and PBD, NR1D1 overexpression. PBD and NR1D1 activity is aimed at the regulation of FAS (fatty acid synthase), ACLY (ATP citrate lyase), and ACACA (acetyl-coenzyme A carboxylase α) gene expression [162,163]. In this regard, palmitate, the last metabolic product of the fatty acid synthesis pathway, was described as lipotoxic agent, likely by ROS induction [164]. Some researchers identified a protective role of PPARγ against palmitate-induced lipotoxicity in ERBB2-positive breast cancer cell lines (BT474 and MDA-MB-361), but not in other types of breast cancer (MCF-7) and normal cells. The PPARγ activity allowed the induction of triacylglycerol synthesis, in order to remove the excess of fatty acid and enclose them in specific stores (LDs) to relieve lipotoxicity. Moreover, PPARγ played a central role in keeping the FAS active by confinement of palmitate in specific stores. The inhibition of PPARγ by antagonist abolished the protective mechanism, and ERBB2 cells were more sensible to palmitate-dependent toxicity (Figure 1B) [165]. A pertinent work showed the suppressive effects of PPARγ antagonism in populations of cancer stem cells (CSCs) derived from ERBB2-positive breast cancer cell lines (BT474 and SKRB3). These cell lines expressed high levels of ALDH (aldehyde dehydrogenase) activity with greater lipid storage than ERBB2-negative cells. Also in this case, the tumor suppressive effects were related to increased ROS levels and a damaged lipogenesis pathway. The researchers’ assumption was that the epigenetic pattern of ACLY (ATP citrate lyase) could be altered by PPARγ inactivation, considering the ACLY gene a PPARγ target gene. In fact, acetylation levels of H3 and H4 histone were found to be different between ERBB2-positive cells and control cells [166].

Recently, an interesting approach, called sleeping beauty (SB), was used to mainly find genes leading to prostate cancer metastatic events. Briefly, this approach is based on transposons, which can induce somatic mutations, and the expression of transposase enzymes could be tissue-specific or ubiquitous [167]. Most of the analyses were conducted on PTEN-null mice, because patients with poor prognosis presented low PTEN levels and conversely high PPARγ and FAS levels. Noteworthy, the insertion of mutations within the PPARγ gene established greater tumor aggressiveness in PTEN-deleted mice than in mice without insertion. Also in this study, the PPARγ overexpression determined the upregulation of enzymes involved in de novo fatty acid synthesis, and conversely, this effect was abolished by PPARγ knockout and downregulation [168]. 

Tumor-associated macrophages (TAMs) have a close relationship with the tumor microenvironment and encourage tumor progression. Several evidences support the idea that stromal cells play a key role in tumor maintenance, since tumor cells exploit them by using their energy resource, in the form of metabolic intermediates or end products (lactate, ketones, glutamine, and fatty acids). Concerning this scenario, the ability of caspase-1 to cut PPARγ in a 41-kDa fragment was reported. Afterwards, this fragment translocates into mitochondria to dampen MCDA activity. Medium-chain acyl-CoA dehydrogenase (MCDA) contributes to fatty acid β-oxidation [169]; its inactivation was demonstrated to be linked to lipid synthesis, and LDs increase with concomitant TAMs differentiation. Considering the caspase-1/PPARγ/MCDA axis as an important mechanism to improve TAMs differentiation and tumor aggressiveness, when this axis was damaged with a caspase-1 inhibitor, TAMs cells suffered a specific commitment that negatively affected tumor progression (Figure 1B) [170]. 

### 2.3. PPARβ/δ and Cancer Metabolism

PPARβ/δ, similar to other PPAR isotypes, regulates the transcription of genes that are required for the main metabolic processes, such as glucose and fatty acid catabolism, although its regulatory role is also implicated in cell proliferation, cell differentiation, wound healing, and inflammation [55,171,172]. Several scientific evidences reported the pro-tumorigenic role of PPARβ/δ, but to date, there has been conflicting information on the exact role of PPARβ/δ in carcinogenesis [75,173]. This aspect was especially investigated in breast cancer with conflicting results, showing that the estrogen receptor was involved in the effects induced by PPARβ/δ activity modulation. In fact, proliferation in the MCF-7 cell line (estrogen receptor positive, ER^+^) was increased by PPARβ/δ overexpression; conversely, the MDA-MB-231 cell line (estrogen receptor negative, ER^−^) showed no effect on the cell proliferation rate. Unfortunately, these results are not consistent with other evidences, showing that in MCF-7 cells, the overexpression of PPARβ/δ induced differentiation and cell cycle interruption [174,175,176]. On the other hand, the negative effect of PPARβ/δ activation on tumor survival in MCF-7 and MDA-MB-231 cell lines has also been reported [177].

Tumor progression in non-small cell lung cancer (NSCLC) was associated with PPARβ/δ upregulation, an increase in VEGF (vascular endothelial growth factor) levels and activation of the PI3K/Akt pathway [178]. PPARβ/δ could be considered an upstream regulator of PI3K/Akt activity. PI3K/Akt signaling is able to reduce PTEN levels and increase PDPK1 (3-phosphoinositide-dependent protein kinase-1) expression [179]. Since the PDPK1 gene presents PPRE specific for PPARβ/δ, as already demonstrated [180], an interesting analysis was conducted on mammary tumorigenesis in an in vivo model. In this regard, transgenic mice carrying the PDPK1 gene under the transcriptional control of mouse mammary tumor virus (MMTV-mice) were used. Nevertheless, the expression was limited to the mammary gland [181]. Transgenic mice showed higher PPARβ/δ expression levels than control mice; the expression was further increased in MMTV-mice fed a diet containing PPARβ/δ agonist. Mammary carcinogenesis was promoted in both wild-type and transgenic mice under feeding treatment, especially in transgenic mice. The researchers emphasized the differences between wild-type and MMTV mice regarding the treatment response, because mice bearing the PDPK1 transgene and treated with PPARβ/δ agonist were more prone to tumor initiation, which might have been due to differences in the involved metabolic pathway. Regarding that, the PI3K/Akt pathway is able to phosphorylate and activate ATP citrate lyase; simultaneously, PDPK1 slows down the pyruvate flow into oxidative phosphorylation and the Acss2 (Acyl-coenzyme A synthetase short-chain family member 2) supports the conversion of lactate to pyruvate. These three proteins work in concert to raise the acetyl-CoA amount in order to promote glycolysis and fatty acid synthesis, and the PPARβ/δ agonist increases their efficiency. Although PDPK1 expression alone was not able to induce carcinogenesis, its association with the active PPARβ/δ triggered a malignancy molecular pathway that was more aggressive in transgenic mice than in wild-type mice treated with PPARβ/δ agonist. Therefore, two different metabolic mechanisms can be activated, whereby PDPK1 induces the expression of PPARβ/δ and vice versa; this loop in turn supports the transcription and the activity of genes related to glycolysis and lipid synthesis. Fatty acid synthesis could be useful for supplying PPARβ/δ endogenous ligands and continuing to feed PDK1-PPARβ/δ loop activity (see also Figure 1C) [181].

Despite the maintenance of hematopoietic stem cells (HSCs) and endurance of muscle cells establishing an unfavorable metabolic condition, they are safeguarded through PPARβ/δ activity. It is likely that PPARβ/δ triggers specific molecular mechanisms related to the metabolic switch to allow the cell life cycle [182,183]. As already demonstrated by Tung and collaborators [117], PPARβ/δ transcription was promoted when leukemic cells were stressed by glycolysis inhibitors. The same results were obtained in a recent paper, but in breast cancer cell lines. When the cells grow in standard culture conditions for 10 days without medium replacement, the overexpressing-PPARβ/δ cells continued to proliferate much better than control cells. Conversely, cells with the PPARβ/δ knockdown, through CRISP/Cas9, showed a proliferation rate comparable to the control levels. However, the low glucose culture conditions induced a more pronounced PPARβ/δ upregulation in transfected cells compared to standard culture conditions, confirming the central role of PPARβ/δ in tumor metabolic modulation. Furthermore, these events were associated with increased levels of catalase and Akt protein, as well as an upregulation of the antioxidant defenses (Figure 1C) [184].

As mentioned above, PPARβ/δ plays, in concert with FAO, a key role in the preservation of HSCs, also in the presence of harsh environmental conditions. Regarding that, PPARβ/δ-FAO pathway undergoes an upstream regulation by the PML (promyelocytic leukemia) protein; which is codified by a tumor-suppressor gene. For example, Ito and colleagues [183] demonstrated that HSCs with *Pml* gene deletion were less inclined to asymmetric division with significant variation of the asymmetric/symmetric division ratio, and there are similar results also in breast cancer cells that sustain this observation [185]. These evidences provide further support regarding PPARβ/δ-FAO pathway regulation by PML upstream control. Therefore, abolishing the oxidative metabolism of fatty acids could damage cancer stem cells and more differentiated scaffold cells [183]. In this regard, a similar effect was observed in chronic lymphocytic leukemia (CLL) cells (Daudi cell line and primary culture), where the stressful environmental conditions stimulated PPARβ/δ expression by triggering a protective mechanism in cancer cells. Various kinds of harsh conditions were tested: low glucose, hypoxia, exposure to glucocorticoids, and cytotoxic agents. In any case, the response of tumor cells was to improve antioxidant activity and make better use of energy supplies through a proper metabolic pathway [186]. More recently, the involvement of PPARβ/δ signaling in the survival of CLL cell lines was reported. This event was associated with increased cholesterol and plasma membrane biosynthesis. Exposure to PPARβ/δ agonists was found to induce high cholesterol levels and interferon-dependent STAT phosphorylation. Cytokines stimulated the specific pathway related to cholesterol synthesis, while the inability of cytokines to upregulate PPARβ/δ was also demonstrated. On the other hand, PPARβ/δ could stimulate the cytokines expression in order to maintain the tumor microenvironment (as reported in Figure 1C) [187].

Consistent with these results, the direct role of PPARβ/δ in the IL-8 gene transcription was also observed in colon cancer cells, mainly in a hypoxic environment [188]. Unlike PPARα and γ, which present both pro-tumor and anti-tumor effects in colorectal cancer, different experimental evidences showed the pro-tumorigenic role of PPARβ/δ, mainly through its involvement in the APC/β-catenin/K-Ras oncogenic pathway [189,190]. The upregulation of PPARβ/δ was observed in human HCT116 colon cancer cells in a hypoxic environment. Whereas p300/PPARβ/δ interaction was triggered by HIF-1. p300 is an all-purpose co-activator for the nuclear receptor that contributes to the formation of transcriptional complex. The authors reported high levels of tumor angiogenesis by IL-8 and VEGF overexpression linked to hypoxic conditions that in turn induce the p300/PPARβ/δ complex. This complex strongly affected the expression of inflammatory cytokines. At the same time, PPARβ/δ is upstream regulated by PI3K/Akt, but PPARβ/δ itself is able to regulate PI3K and Akt expression; thus, a permanently active closed loop could be generated, as indicated in Figure 1C [188].

As mentioned above, PPARγ is directly involved in the differentiation of TAMs [170]; it is also worth mentioning that macrophages can assume two specific phenotypes: M1 (inflammatory) and M2 (anti-inflammatory). However, TAMs present a mix of both phenotypes [191]. PPARγ and PPARβ/δ are able to regulate the final fate of macrophages in a tumor environment. The transcriptional control of PPARβ/δ on genes linked to TAMs was observed in ovarian cancer cells. CD14^+^ monocytes cells from ovarian carcinoma ascites were used as TAMs in vitro model [192]. This study evaluated which genes related to TAMs were under PPARβ/δ transcriptional control. The overall results confirmed the regulation of metabolic pathway genes and inflammatory/migration pathway genes. The upregulation of these genes was also found in the presence of PUFA (poly-unsaturated fatty acid) ligands. Therefore, transcriptional regulation by PPARβ/δ could be associated not only with the maintenance of TAMs, but also with tumor progression. The upregulation of genes encoding for soluble mediators of cancer progression, such as ANGPTL4 (angiopoietin-like 4), could be under the transcriptional control of the PPARβ/δ. ANGPTL4 is a lipoprotein lipase regulator; it is essential for tumor-metastatic progression. In fact, angiopoietin-like 4 prevents the cell death by anoikis [193,194]. However, the preservation of TAMs was dependent of PPARβ/δ activation, which in turn induced the transcription of downstream elements, such as ANGPTL4 and PDK4, in order to allow a metabolic switch to aerobic glycolysis (Figure 1C). In fact, high lactate levels were detected, and cells from ascites resulted in having high fatty acid ligands for PPARβ/δ; thus, the nuclear receptor activity in TAMs maintenance was greatly facilitated by the tumor microenvironment [192].

Since the metabolic fate undertaken by cancer cells is a response depending on the cell phenotypic/genotypic characteristics, and on the specific microenvironment around the neoplastic bulk. PPARβ/δ behavior also undergoes this specific tumor conditioning. However, the microenvironment affects the ability of cancer cells to acquire nutrients from extracellular compartments to cytoplasm by transmembrane transporter proteins. In this regard, Zhang et al. [77] reported the direct binding of PPARβ/δ on the PPRE of *Glut1* and *Slc1-a5* genes, and highlighted their upregulation by PPARβ/δ activation, in order to ensure glucose and amino acids for tumor growth. Transfected SW480 cells (cell line from colon adenocarcinoma) with PPARβ/δ transgenes showed high mRNA and protein levels of GLUT1 and SLC1-A5 (solute carrier family 1 member 5), and as a consequence, lactate increases, and there is also high glucose and glutamine consumption. All of these results were abolished by PPARβ/δ knockdown or through the use of an antagonist. Moreover, the overexpression of GLUT1 and SLC1-A5, with contemporary PPARβ/δ silencing, caused an increase in the proliferation rate, which was abolished in cells with a specific deletion of the transporter protein genes and overexpression of PPARβ/δ. Considering these results, it is conceivable to hypothesize that there is a PPARβ/δ-dependent molecular pathway leading to GLUT1 and SLC1-A5 upregulation, resulting in the modulation of metabolic patterns suitable for tumor growth (Figure 1C) [77].

Unlike the evidence reported so far, the oncosuppressive activity of PPARβ/δ in prostate cancer was recently demonstrated [195]. In a tumor tissue biopsy of prostate cancer, low mRNA levels of PPARβ/δ were observed compared with benign tissue. The same results were obtained in prostate cancer cell lines (DU145, PC3, LNCAP, VCAP, C4-2, and 22RV); thus, the downregulation of PPARβ/δ was associated with high aggressiveness. In the absence of ligands, the PPARβ/δ could exist as a transcriptional repressor [172]; in fact, the inhibition of FAO was demonstrated when PPARβ/δ was overexpressed, but only in PC3 and LNCaP cell lines. The repressive effect on FAO was abolished by PPARβ/δ agonist, without affecting PPARβ/δ transcription levels. The results obtained confirm the suppressive role of PPARβ/δ in its unliganded form; see also Figure 2C [195].

## 3. Conclusions

Although there is no clear view on the exact role of PPARs in carcinogenesis, and considering that most of the experimental proofs are mutually conflicting, the key role of PPARs in the metabolic modulation faced by cancer cells to ensure their own survival has been accepted. Each cancer cell exhibits a specific metabolic signature that is related to its tissue-specific genotypic and phenotypic features. Nevertheless, the specific cell phenotype has a close relationship with the microenvironment around the tumor bulk; thus, tumor phenotypic manifestations are the result of the effects induced by the tumor microenvironment on cellular transcription events. Regarding that, the transcriptional activity of PPARs on specific target genes is deeply correlated to the tissue type from which the tumor arises and to the tumor microenvironment. For this reason, each PPAR isotype establishes different effects in various tumor cell types. Overall, all of these factors determine whether PPARs promote tumorigenesis and tumor progression or counteract cancer survival. Moreover, the tumor microenvironment provides PPAR ligands; consequently, the extracellular environment can directly modulate the activities of PPARs.

The most recent evidences reported in this review demonstrate the involvement of PPARs in a metabolic switch that occurs in different cancer types. The oncogenic metabolic pathway of PPARα is characterized by high glycolysis in concert with c-Myc and cyclin D1 upregulation, as well as high levels of lipid and glycogen synthesis. In addition, an increase of LDs is observed that is associated with the upregulation of the MVA pathway, while, less frequently, PPARα oncogenic activity can be connected to the induction of OXPHOS and FAO. Moreover, an increase in fatty acid oxidation was reported to confer chemoresistance, i.e., against glucocorticoids [116]. Hypoxia exerts its oncogenic role through a stimulation of PPARα transcriptional activity (Figure 1A). Oncogenic metabolic behavior related to PPARγ activity mainly triggers an increase in lipid synthesis and reduces FAO. Meanwhile, lipotoxicity related to a high amount of palmitate is arrested by PPARγ, which drives palmitate confinement into LDs. The positive role of PPARγ in the differentiation of TAMs is intriguing; the behavior of tumor stromal cells is affected by the PPARγ-mediated inhibition of FAO and induction of lipid synthesis (Figure 1B). Unlike other two PPAR isotypes, most of the evidence regarding PPARβ/δ activity highlights its oncogenic role. Environmental stress, such as hypoxia and low glucose, triggers the tumor metabolic pathway under PPARβ/δ transcriptional control; thus, aerobic glycolysis, lipid synthesis, anaplerosis, and FAO are stimulated. It is noteworthy in leukemia cells that the upstream regulation of cytokines by PPARβ/δ is related to high cholesterol levels and malignancy (Figure 1C).

Under certain circumstances, the transcriptional activity of PPARs is aimed at suppressing specific tumor metabolic pathways. PPARα can inhibit lipid and cholesterol synthesis in concert with FAO induction. Glycolysis is obstructed by the PPARα-dependent destruction of the hexokinase II/VDAC complex, leading to metabolic switch and high OXPHOS levels, as demonstrated in oral cancer cells (Figure 2A) [127,128]. Unlike PPARα, in some tissues, the hypoxia inducible factor downregulates PPARγ, leading to the loss of its tumor suppression activity. In normoxic conditions, PPARγ represses the expression of the gene related to glycolysis (Wnt/β-catenin, c-Myc), glutamine anaplerosis, chemoresistance, and antioxidant defenses. Conversely, PPARγ transcriptional activity encourages the expression of genes involved in tumor differentiation, TCA cycle, and FAO, which are all in agreement with the PKM1/PKM2 ratio increase (Figure 2B). Among the scant evidence supporting the oncosuppressive role of PPARβ/δ, its ability to decrease FAO and disrupt tumor proliferation in prostate cancer cells is accepted, but only in absence of its ligands (Figure 2C).

Overall, this review highlights the central role of PPARs in tumor metabolic decisions, which are in turn affected by the genetic signature of tumor cells and the specific tumor microenvironment. In this regard, epigenetic events could play a key role in the regulation of PPAR activities in tumor metabolic response, while the possible relationship between the three PPARs isotypes in tumor metabolism should be taken in consideration, as already described in the pathogenesis of neurodegenerative diseases [196]. However, in order to fully understand the exact role of PPARs in cancer metabolism, studying the epigenetic effects related to PPARs and the relationship between the three isotypes could be interesting, in order to efficiently target the complex machinery that achieves the energy demands of cancer cells.
